# An Unusual Finding of a Neurogenic Tumor of the Trachea: A Tracheal Schwannoma

**DOI:** 10.7759/cureus.48172

**Published:** 2023-11-02

**Authors:** Juan D Botero, María Pérez Restrepo, Mauricio A Murillo, Felipe Campo, Luis Correa

**Affiliations:** 1 Pulmonology, Clínica Cardio VID, Medellín, COL; 2 Internal Medicine, Universidad CES, Medellín, COL; 3 Pathology, Clínica Cardio VID, Medellín, COL

**Keywords:** interventional pulmonology, argon laser, schwannoma, bronchoscopy, tracheal neoplasms

## Abstract

We report a rare case of a 57-year-old female patient with intraluminal tracheal obstruction caused by a benign schwannoma. She underwent successful bronchoscopic resection under general anesthesia, with no complications observed during the post-procedure follow-up. Tracheal schwannomas are exceedingly uncommon, and while conventional treatment involves surgical resection, bronchoscopic techniques, such as laser ablation, can be a valuable alternative, particularly for high-risk patients. Further studies are needed to explore the full potential of bronchoscopic interventions in managing tracheal schwannomas.

## Introduction

Here, we present a unique case of an uncommon tracheal lesion, eventually diagnosed as a benign schwannoma, which is a rare tumor originating from myelinating cells known as Schwann cells. Given its exceptional location and the suspicion of it being a benign lesion, the medical team opted for an endoscopic procedure using argon recanalization. Later, it was realized that the etiology of the lesion was a rare case of tracheal schwannoma.

This article delves into the diagnostic challenges, therapeutic decisions, and patient's positive outcomes, highlighting the significance of embracing innovative techniques to manage unique clinical scenarios. By sharing this remarkable case, we contribute to the collective knowledge of pulmonology and underscore the value of multidisciplinary collaboration in advancing patient care for a healthier future.

## Case presentation

**Figure 1:**
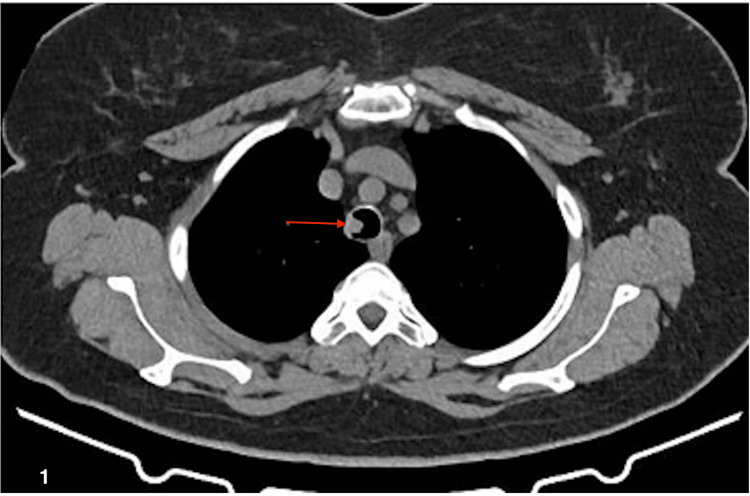
CT scan showing tracheal endoluminal lesion

A 57-year-old female patient was admitted to the pulmonology department due to a three-month history of dyspnea on exertion (MMRC scale 2/4), associated with a persistent dry cough and a weight loss of approximately 5 kg in the past year. She had been evaluated at an outpatient clinic where her primary care physician had performed a chest tomography that showed an endoluminal image in the trachea (see Figure 1). With this result, her primary care doctor suggested a pulmonology consult.

**Figure 2:**
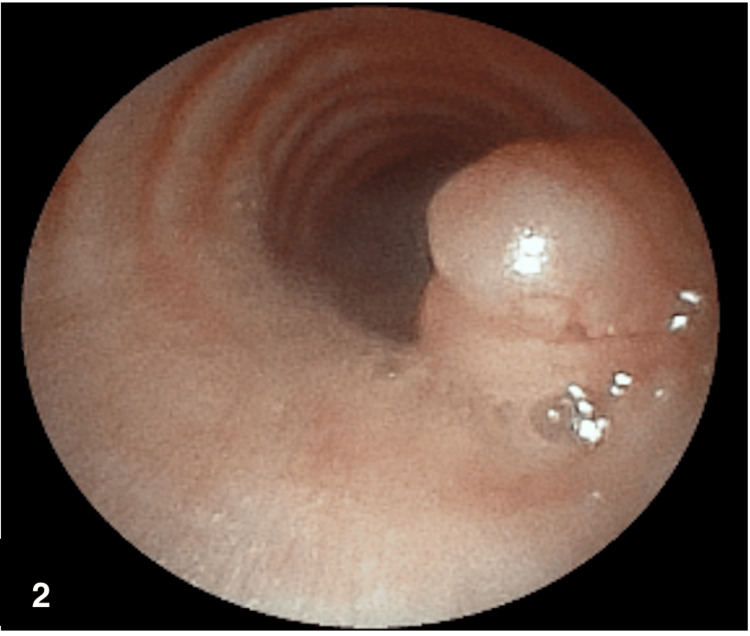
Flexible fiberoptic bronchoscopy revealed an exophytic, irregular, sessile lesion with mucoid appearance and vascularization in the trachea

Bronchoscopy was performed for evaluation and biopsy. An irregular, exophytic, sessile, mucoid, vascularized lesion was identified at 6 cm distal from the vocal cords, with an extension of approximately 1 cm, that occluded 35% of the lumen (see Figure 2).

Samples were taken for microbiology, and seven biopsies were performed, reporting lymphocyte-predominant inflammation, without microbiologic isolates. A contrast-enhanced chest and abdominal tomography was performed on the patient, showing a single lesion, that had not grown in the last three months and without infiltration into deep tissues. Given those characteristics, the absence of initial malignancy on histology, and the possibility of a granulomatous lesion in the trachea, it was decided to schedule an endoscopic resection.

**Figure 3:**
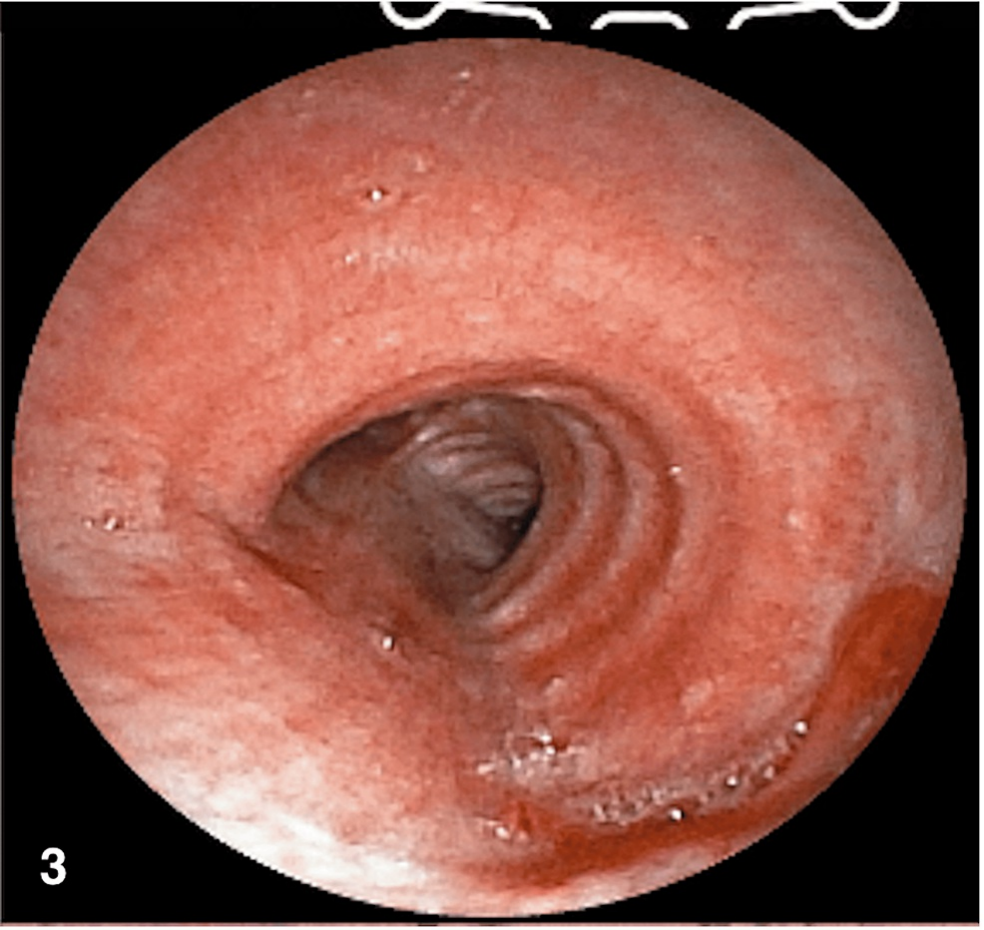
Post resection bronchoscopy

She was taken for bronchoscopy and debulking, a procedure that was performed under general anesthesia, with the use of an N4 laryngeal mask. A Fuji therapeutic bronchoscope was used to recanalize with argon, achieving a complete resection and permeating the lumen (see Figure 3). Her subsequent course was adequate, and she was discharged.

**Figure 4:**
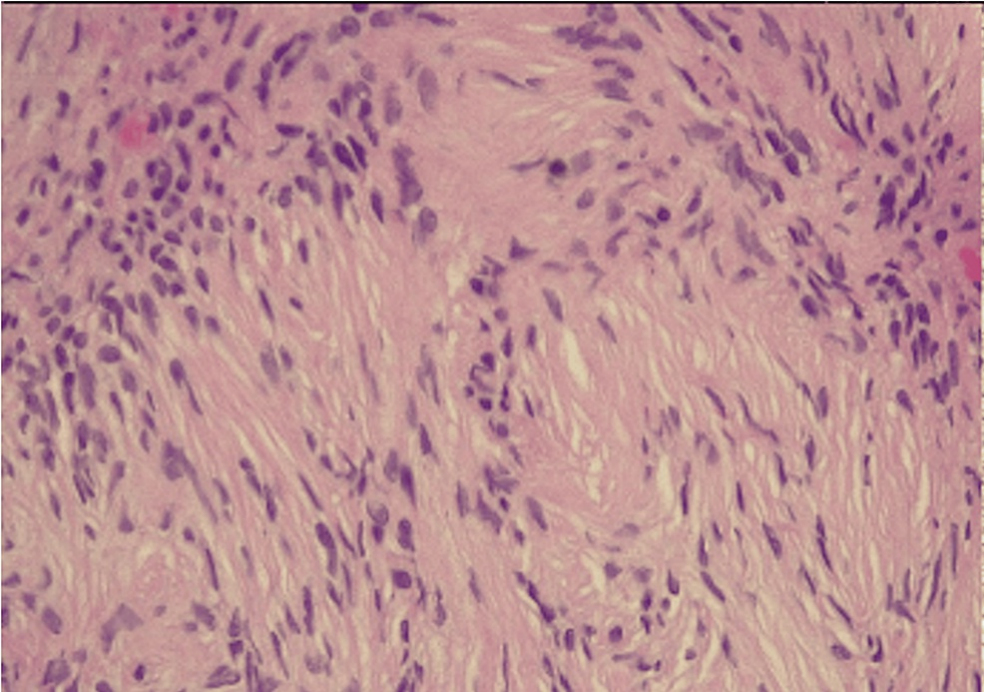
Hematoxylin-eosin staining demonstrates pathology, revealing lymphocyte-predominant inflammatory infiltrate and classifying spindle cell injury

**Figure 5:**
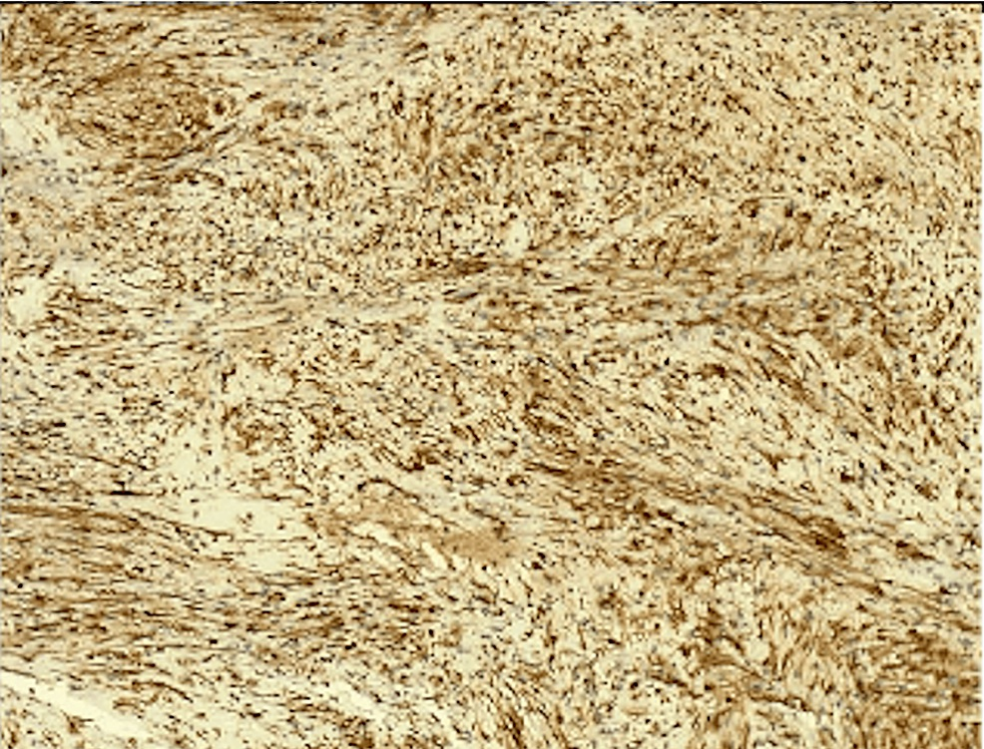
Special stains positive for schwannoma

After discharge, the pathology report revealed a well-limited, benign nerve sheath lesion (see Figure 4), positive for S100 and SOX10 (see Figure 5), findings that support the schwannoma diagnosis. In the follow-up three months after the procedure, the patient is well, and the tomography and bronchoscopy have not shown recurrence.

## Discussion

Intraluminal obstruction of the trachea is a low-prevalence clinical entity that usually has a benign origin. Conversely, primary tracheal tumors are mostly of malignant origin, with a reported prevalence of 0.1 per 100,000 persons/year, representing less than 0.5% of all malignant tumors [1]. The mean age of diagnosis is approximately 60 to 65 years, with a prevalence in males of 60-70% [1]; 44.8% correspond to squamous cell carcinomas and 16.3% to adenoid cystic carcinoma.

Schwannomas are encapsulated tumors of the peripheral nervous system, originating from Schwann cells, which rarely have malignant progression [2]. Between 80 and 90% of these tumors are present at the vestibular level, so their tracheal identification is extremely infrequent, with few cases described in the literature presenting with dyspnea, cough, wheezing, hemoptysis, and sometimes fever caused by associated pulmonary infection [3]. This lesion is mainly diagnosed through tomographic images, with subsequent direct visualization and biopsy for diagnostic confirmation with fiberoptic bronchoscopy.

The standard of treatment described for this lesion is surgical circumferential resection with tracheal reconstruction. However, schwannomas can be treated using bronchoscopic techniques, mainly laser ablation, with great success in completely intraluminal and pedunculated tumors, especially in patients with high surgical risk [4]. In the largest case series published by Jin et al., bronchoscopic management of six patients with tracheal schwannomas was performed, with a mean follow-up of six years, with excellent results [5].

## Conclusions

A case of tracheal lesion initially identified by tomography is presented. After direct visualization with fiberoptic bronchoscopy and biopsy, it was possible to diagnose a tracheal schwannoma, treated with endoscopic management with excellent results.
